# Analysis of exotic squirrel trade and detection of human infections with variegated squirrel bornavirus 1, Germany, 2005 to 2018

**DOI:** 10.2807/1560-7917.ES.2019.24.8.1800483

**Published:** 2019-02-21

**Authors:** Dennis Tappe, Christina Frank, Timo Homeier-Bachmann, Hendrik Wilking, Valerie Allendorf, Kore Schlottau, César Muñoz-Fontela, Monika Rottstegge, Julia R Port, Jürgen Rissland, Philip Eisermann, Martin Beer, Jonas Schmidt-Chanasit

**Affiliations:** 1 Bernhard-Nocht-Institut für Tropenmedizin, Hamburg, Germany; 2 Robert Koch-Institut, Berlin, Germany; 3 Friedrich-Loeffler-Institut, Greifswald/Insel Riems, Germany; 4 Institut für Virologie, Universitätsklinikum des Saarlandes, Homburg/Saar, Germany

**Keywords:** exotic pets, animal trade, variegated squirrel bornavirus 1, case definition, serology, epidemiology, viral infections, encephalitis, viral encephalitis, surveillance, laboratory

## Abstract

Following the discovery in 2015 of the variegated squirrel bornavirus 1 (VSBV-1) in fatal encephalitis cases among exotic squirrel breeders and a zoo animal caretaker in Germany, a case definition was developed. It was employed during trace-back animal trade investigations and sero-epidemiological studies among breeders and zoo animal caretakers of holdings with VSBV-1 infected squirrels. During the investigation, two possible human cases who had died of encephalitis were identified retrospectively among the squirrel breeders. Moreover, one probable human case was detected among the breeders who had a positive memory T-cell response to VSBV-1 antigen and antibodies against VSBV-1. The low rate of seropositivity found among living persons in risk groups that handle exotic squirrels privately or at zoos may reflect rareness of exposure to VSBV-1 during animal contact, a high lethality of infection or a combination of these factors. As a precaution against human exposure, testing of exotic squirrels for VSBV-1 infection and/or avoiding direct contact with exotic squirrels in zoos and private holdings is strongly advised.

## Background

In 2015, the variegated squirrel bornavirus 1 (VSBV-1, species *Mammalian 2 orthobornavirus*) was discovered as an emerging zoonotic pathogen responsible for three cases of fatal encephalitis among private breeders of exotic squirrels in Germany [1]. All three were male, resident in the state of Saxony-Anhalt (Cases A, B, C) and had succumbed to encephalitis between 2011 and 2013. All had kept variegated squirrels (*Sciurus variegatoides*), an exotic squirrel species from Central America. Recently, in early 2018, the virus was retrospectively detected also in the brain of a zoo animal caretaker from the German state of Schleswig-Holstein (Case D). This female patient had had occupational contact to an infected Prevost’s squirrel (*Callosciurus prevostii*), an exotic species from Southeast Asia. She had died of limbic encephalitis in 2013 [2]. VSBV-1 has been tentatively classified as a biosafety level 3 pathogen. The incubation time and the mode of transmission have not been determined. Animal scratches and bites are considered the main risk factor [1].

Following the retrospective detection in 2015 of the VSBV-1 encephalitis cluster among the breeders of variegated squirrels [1], the European Centre for Disease Prevention and Control (ECDC) issued a rapid risk assessment. It stressed the need for investigations into the natural hosts, reservoirs and transmission route of the virus owing due to the novel nature of the event [3]. As a precautionary measure, direct contact to exotic squirrels should be avoided. Since then, molecular testing for VSBV-1 infection among exotic squirrel species has been performed in Germany in private holdings and zoos and has been recommended as a standard measure to reduce the risk of human infection. VSBV-1-positive exotic squirrels and/or humans were identified at seven private holdings (including the holdings of Cases A, B and C) and four zoos (including the zoo where Case D had worked) between 2015 and 2017 [4,5]. Testing of exotic squirrels in Germany is continuously ongoing.

Here, we report an epidemiological investigation of the exotic squirrel trade between private holdings, the detection of additional human fatalities linked to VSBV-1 among squirrel breeders, and a serological study of past VSBV-1 infections among private squirrel breeders and zoo animal caretakers in Germany.

## Methods

### Epidemiology and human case finding

Trace-back interviews about exotic squirrel trading were conducted between 2015 and 2018 in the private holdings of the three squirrel breeders who had died of VSBV-1 encephalitis [1] and in four further holdings where VSBV-1-infected squirrels had been found previously [4,5]. We contacted squirrel breeders and holders identified by the tracing of the animal trade, as well as their household members, by telephone or email. In systematic questionnaire-based interviews, the participants were asked about the history of their holdings, their general husbandry management, trading contacts and animal exchange with others (squirrel species, year of trade), and rate of contact to the animals (regularly, occasionally, rarely). We identified contact holdings and offered them diagnostic testing of their squirrels, accompanied with a questionnaire-based interview. Because there was no registry of squirrel owners or documentation on the individual animal exchanges between the holdings, the data on the trading contacts were based on the participants’ memories. Interview participants were also asked to take part in a serosurvey for past VSBV-1 infection.

Zoos were informed, by the federal states’ authorities and by scientific lectures at meetings of the German Zoological Society, about the discovery of VSBV-1 and the possibility to have their animals tested. In all four German zoos where VSBV-1-positive Prevost’s squirrels had already been found [4,5], the respective physicians responsible for the zoo workers were informed and the zoo animal caretakers were asked to participate in the serological study. 

Human case finding was performed among the squirrel breeders and zoo animal caretakers, employing a graded case definition (confirmed, probable and possible; Box). Encephalitis or encephalopathy were defined according to Venkatesan et al. [6].

Box **Case definition for acute human infections caused by the variegated squirrel bornavirus 1, Germany, 2015–2018**

**Table:** 

**Confirmed case of VSBV-1 infection**
Newly developed encephalitis or encephalopathy AND Detection of VSBV-1 RNA in CSF or CNS tissue OR Detection of seroconversion in serum or CSF with IgG antibodies against VSBV-1 by screening test (e.g. IFAT) and confirmation assay (e.g. ELISA or immunoblot) OR ≥ 4-fold increase of antibody titres in follow-up samples of serum or CSF with IgG antibodies against VSBV-1 by screening test (e.g. IFAT) and confirmation assay (eg. ELISA or immunoblot)

**Probable case of VSBV-1 infection**
Newly developed encephalitis or encephalopathy AND Detection of IgG antibodies against VSBV-1 in a serum or CSF sample by screening test (e.g. IFAT) and confirmation assay (e.g. ELISA or immunoblot) AND No evidence of other reasons for the clinical picture

**Possible case of VSBV-1 infection**
Newly developed encephalitis or encephalopathy AND Contact to exotic squirrel species AND No evidence of other reasons for the clinical picture

CNS: central nervous system; CSF: cerebrospinal fluid; ELISA: enzyme-linked immunosorbent assay; IFAT: immunofluorescence antibody test; VSBV-1: variegated squirrel bornavirus 1.

### Serological testing of humans for past infection with variegated squirrel bornavirus 1

For screening and confirmation of past human VSBV-1 infection, we employed newly developed enzyme-linked immunosorbent assays (ELISAs) and immunoblot assays with recombinant VSBV-1 N and P proteins [2], as well as an indirect immunofluorescence antibody test (IFAT) using a Borna disease virus 1 (BoDV-1)-infected cell line [2]. 

### Flow cytometry analyses

In seropositive persons, we conducted flow cytometry analyses for the detection of memory T-cell responses to VSBV-1. Bioinformatic analyses were performed to determine immunodominant VSBV-1 peptides predicted to bind to the patient’s phenotypically determined human leukocyte antigen (HLA) type with high affinity. An artificial neural network method at the Immune Epitope Database and Analysis Resource (IEDB) (www.iedb.org) was used for the selection of suitable peptides. Selected peptides (half-maximal inhibitory concentration IC50 < 50 nM) were cross-checked with two additional matrix prediction algorithms, BIMAS (http://www-bimas.cit.nih.gov) and SYFPEITHI (http://www.syfpeithi.de). Peptides predicted by all three bioinformatic tools were selected and screened for similarity to the human genome using the National Institutes of Health (NIH) Blast server. Peptides showing homology to the human proteome were discarded. We selected the peptide FLCLLIPGL for dextramer design and used custom dextramers conjugated with allophycocyanin (APC) (Immudex, Copenhagen, Denmark). 

T-cell phenotype was assessed by flow cytometry. Briefly, peripheral blood mononuclear cells were isolated from EDTA blood. Identification of T-cell subsets was achieved by a multiparametric panel using commercially available antibodies (anti-CD3, clone UCHT1; anti-CD4, clone OKT4; anti-CD8, clone RPAT8; anti-CD56, clone 5.1H11; anti-CD45R/B220, clone RA3–6B2; anti- CCR7, clone G043H7; anti-CD45RA, clone HI10). In order to determine the homing profile of T-cells, antibodies against beta 7 integrin (clone FIB27) and cutaneous lymphocyte antigen (CLA, clone HECA-452) were included. All antibodies were purchased from BioLegend (San Diego, United States (US)). Sample acquisition was done in an LSR Fortessa instrument (Becton Dickinson, Heidelberg, Germany). Flow cytometry analysis was performed with FlowJO software (Treestar, Ashland, US).

### Ethical statement

There was no need for ethical approval. Written consent was obtained from all participants of the serological study and from next of kin of those who had died. 

## Results

### Interviews

In total, all 20 squirrel breeders and holders who could be contacted took part in the interviews. Based on those, we found that *S. variegatoides* was initially imported from Costa

**Figure 1:**
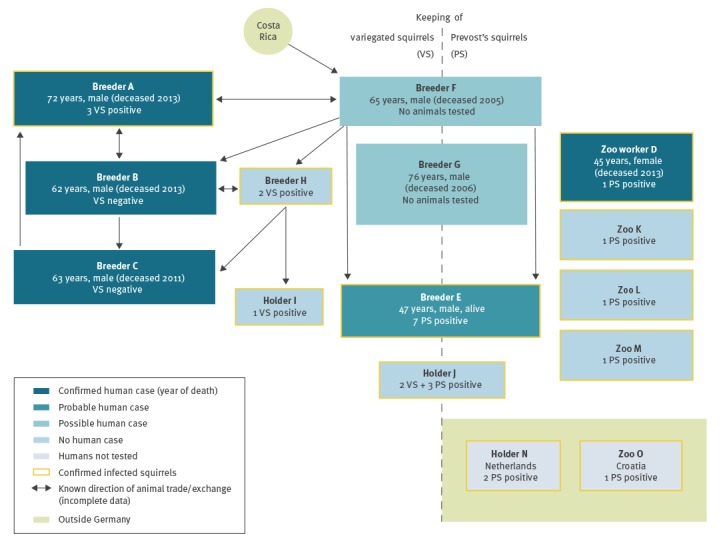
Known animal traffic among holdings with variegated squirrel bornavirus 1-positive exotic squirrels, Germany, 2015–2018

Rica to Germany in 1999. *C. prevostii* was introduced to Germany earlier, in the 1980s, from Southeast Asia (i.a. Malaysia and Thailand). Many breeders kept both species, and physically close to one another. Breeders and holders were regularly exposed to the squirrels, whereas household members had only rare or occasional contact to the animals. From the study participants’ answers, parts of a complicated squirrel trading network were generated, including the seven holdings with human and/or squirrel VSBV-1 cases (Figure 1).

Positive/negative: variegated squirrel bornavirus 1 test result; PS: Prevost’s squirrel (*Callosciurus prevostii*); VS: variegated squirrel (*Sciurus variegatoides*).

Based on positive VSBV-1 PCR results in squirrels, an interconnected trading network is depicted, including the case definition status of the breeders and holders. The affected humans are from five different federal states in Germany. The affected zoos and holdings with only positive animals are located in six (four additional) federal states and two other European countries. Holdings known to have kept both squirrel species at some point are placed on the dotted dividing line. Arrows symbolise the direction of animal traffic. Animal traffic from 2005 until the detection of the first human VSBV-1 encephalitis cases in 2015 is shown.

In the course of the squirrel trade investigation, we identified two male squirrel breeders who had died of encephalitis of unknown causes in 2005 (Case F) and 2006 (Case G) in the federal states of North Rhine-Westphalia and Brandenburg, respectively. These men in their 60s and 70s had developed fatal meningoencephalitis (death after 1 year in one patient, and after 1 month of illness in the other). The patients had underlying cystic renal disease with hyperuricaemia and chronic obstructive pulmonary disease, respectively. Symptoms had consisted of initial fever and cognitive dysfunction in both, followed by facial paresthesia, dysarthria, dysphagia, tetraparesis, and focal seizures in Case F, and myoclonus, opisthotonus and coma in Case G. Cranial magnetic imaging had revealed basal ganglia inflammation and analyses of cerebrospinal fluid had shown lymphocytic pleocytosis. During initial attempts to identify the disease aetiology, no known pathogens had been detected. Autopsy conducted for Case G showed basal ganglia and brain stem inflammation. 

Retrospective laboratory examination for VSBV-1 could not be conducted: no samples had been stored from Case F and the tissue blocks from Case G had been discarded in 2016. Case F had kept variegated squirrels after direct import from Costa Rica since 1999, Case G had kept variegated squirrels since 2001. Both cases had kept Prevost’s squirrels since 2001. According to the proposed case definition, these two individuals were classified as possible cases.

### Serosurvey and flow cytometry analyses

Fourteen of 20 contacted breeders and holders of exotic squirrels (including all private holdings where VSBV-1-infected squirrels were found previously) and nine of their household members took part in the serosurvey. Among the 14 breeders and holders (age range: 46–65 years; nine men, five women) and nine household members (including households of Cases A, B, C, E (see below), F and G), we identified one seropositive individual, a squirrel breeder (Case E). The man was in his 40s, from the federal state of Lower Saxony, had no medical preconditions and tested positive for IgG against VSBV-1 by IFAT (titre: 1:320), immunoblot (VSBV-1 N antigen) and ELISA (VSBV-1 N antigen). In 2004, he had developed fever for approximately 2 weeks, followed by headaches, lethargy and myoclonus for about 1 year without visible lesions on cranial computed tomography. He had fully recovered. 

**Figure 2:**
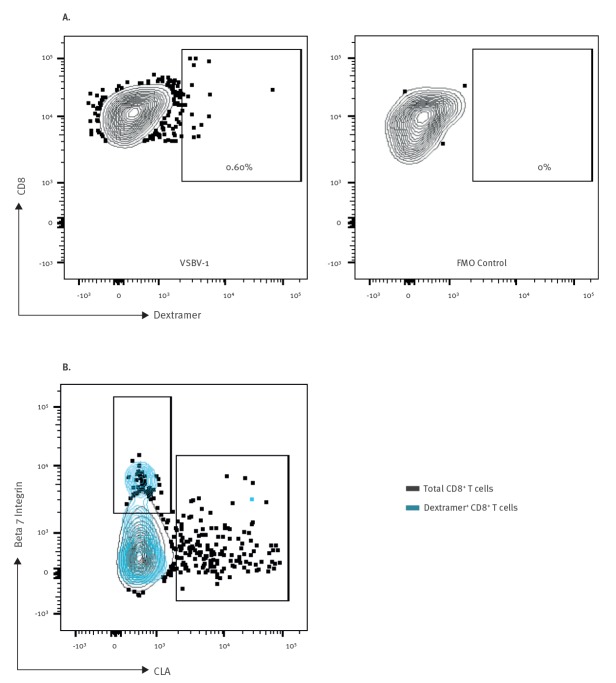
Identification of variegated squirrel bornavirus 1-specific CD8^+^ T-cells in a seropositive individual, Germany, 2015–2018

CLA: cutaneous lymphocyte antigen; FMO: Fluorescent minus one; VSBV-1: variegated squirrel bornavirus 1.

The patient has been keeping several exotic squirrel species since 1993. In his holding, seven VSBV-1-positive Prevost’s squirrels were found during two examinations between 2015 and 2016 [5]; he had also kept variegated squirrels until 2009. We considered this man a probable case. Analysis of his peripheral blood CD8^+^ T-cells (HLA-A*02:01 type) by flow cytometry showed positive dextramer staining of a subset of cells, suggesting the presence of VSBV-1-specific memory T-cells. Evaluation of tissue homing factor expression in dextramer-positive cells revealed the presence of cells with surface expression of beta 7 integrin (Figure 2), a marker of lymphocyte homing to the mucosa.

A. Dextramer-positive CD8^+^ T-cells were identified by flow cytometry. Cells were gated as singlets, CD3^+^ CD56/CD19^−^ cells. FMO control is shown.

B. Dextramer-positive cells were plotted over total CD8^+^ T-cells expressing homing factors for the mucosa (B7 integrin) and skin (CLA).

Twenty-four zoo animal caretakers from three of four zoos in Germany that had kept VSBV-1 infected exotic squirrels took part in the serosurvey. In the fourth zoo, the workplace of Case D, a serosurvey among the zoo workers has been conducted recently as part of a different study, without clear evidence of past VSBV-1 infection [2]. Among the 24 zoo animal caretakers (age range: 20–56 years; seven men and 17 women), no seropositive individuals were detected in the three zoos investigated here.

## Control measures

As a precaution against human exposure, avoiding direct contact with all exotic squirrel species in private holdings and in zoos was after discovery of the first cases strongly advised by the ECDC, the Robert Koch Institute, the Friedrich Loeffler Institute and local veterinary health authorities [3,7,8]. The death from VSBV-1 of a zoo animal caretaker in 2013 was only discovered in early 2018 [2] after an infected squirrel in that zoo was found during the testing campaign of exotic squirrels for VSBV-1 infection in Germany between 2015 and 2017 [4,5]. This finding underscores the necessity of testing all exotic squirrels for VSBV-1 infection (mouth swabs and faecal sampling for RT-qPCR) as currently recommended by the Friedrich Loeffler Institute [9]).

## Discussion

Our report highlights the complexity of exotic animal trade networks and the difficulties in conducting epidemiological investigations in such specific trade communities. Contacting the private squirrel breeders/holders was difficult as there is no registry of squirrel breeders in Germany. Moreover, not all private breeders were willing to participate in the human seroprevalence survey. As the story unfolded over time, more visits per site were necessary. Tracing back the animal trade proved difficult and only limited information on animal exchange could be gathered from the squirrel breeders. For example, Case G and Holder J, as well as the zoo holdings, could not be tied to other holdings with positive squirrels. How the private squirrel trade might have been interconnected with the zoo animal trade could not be elucidated.

In addition to four confirmed fatal cases of human VSBV-1 infection that we had detected previously [1,2], we here retrospectively identified one probable and two possible human cases of past VSBV-1 infection in Germany. Of those seven cases, five (all in their 60s and 70s) had underlying medical diseases, whereas two (in their 40s) had been previously healthy. Possible Cases F and G had died of meningoencephalitis 5–8 years before the confirmed cases. In the surviving probable Case E, IFAT, ELISA and immunoblot testing demonstrated an antibody response against VSBV-1. Antibodies reacted against the VSBV-1 N protein and not against the P protein by ELISA and immunoblot, probably reflecting past VSBV-1 infection. For comparison, detection of antibodies in animals against BoDV-1 N protein and the absence of antibodies against BoDV-1 P protein signals past infection with the closely related BoDV-1 [7]. In contrast, during acute human encephalitis caused by VSBV-1, IFAT titres are much higher and antibodies against both N and P proteins are detectable by ELISA and immunoblot [2]. In this person, we could also measure the presence of CD8^+^ T-cells specific for the VSBV-1 N protein, suggesting exposure followed by establishment of virus-specific memory T-cell responses. Moreover, homing factor analysis revealed the mucosa as a putative site of exposure to VSBV-1 in this individual, rather than animal scratches or bites.

Infected squirrels do not show any VSBV-1-related clinical symptoms but harbour high viral loads in their central nervous system and in organs capable of secretion and excretion (kidneys, urinary bladder, skin and oral cavity); they are thus probably a natural reservoir [1,4,5]. Despite a considerable rate of VSBV-1 infections among exotic squirrel species (*Callosciurinae*: 8.5%; *Sciurinae*: 1.5%) in zoos and private holdings [4], only one seropositive exposed human was found among 49 persons tested in our study. No seropositive individuals were found among household members of Cases A, B and C, and zoo animal caretakers in zoos with infected squirrels. The low rate of seropositivity among living persons in the risk groups that handle exotic squirrels privately or at zoos may reflect rareness of exposure to VSBV-1 during animal contact, a high case fatality (only one case in a currently known total of seven possible, probable and confirmed cases has survived) or a combination of these factors. More animal testing and more human seroprevalence studies, as well as an increased awareness of acute VSBV-1 encephalitis cases are needed, also in other European countries where infected exotic squirrels had been detected (e.g. the Netherlands and Croatia, where Holder N and Zoo O were identified). Furthermore, the geographic origin of the virus is as yet unknown. As exotic squirrels of both the Central American *Sciurinae* and the Southeast Asian *Callosciurinae* subfamilies were found to be infected and were often kept together in German holdings [4,5], a Central American, Southeast Asian or European (German) origin of VSBV-1 appears possible. However, German red squirrels (*Sciurus vulgaris*) have so far not been found to be infected [4].

Recently in 2018, a cluster of organ transplant-associated human encephalitis cases with two fatalities caused by BoDV-1, and a non-transplant-associated fatal BoDV-1 encephalitis case were described in Germany [11,12]. Currently, seroprevalence studies, retrospective case finding studies and risk factor analyses for human BoDV-1 encephalitis are being performed in Germany. Previous attempts to link human BoDV-1 infections to neuropsychiatric illness have not resulted in conclusive evidence [13].

## Conclusion

Our study highlights the human health threats that can emerge from uncontrolled exotic pet animal trading. In the recent past, there have been reports of severe human infections such as monkeypox from prairie dogs that had acquired the virus from imported giant rats [14], cowpox from pet rats [15,16], Seoul hantavirus infections from pet rats [17,18] and salmonellosis from pet turtles and other reptiles [19,20]. Following the One Health concept, virologists, epidemiologists and public health specialists from human and veterinary medicine were involved in our study to investigate a novel zoonosis.
